# Involuntary Admissions and Patient Autonomy - How do they Fit Together

**DOI:** 10.1192/j.eurpsy.2022.132

**Published:** 2022-09-01

**Authors:** M. Schouler-Ocak

**Affiliations:** Charité – Universitätsmedizin Berlin, Psychiatric University Clinic At St. Hedwig Hospital, Berlin, Germany

**Keywords:** patient autonomy, patient-controlled admission, ethical aspects, Involuntary admission

## Abstract

The United Nations Convention on the Rights of Persons with Disabilities Article 12 General Commentary, explicitly states that persons with mental illnesses must always have full exercise of their legal rights in all their aspects. Assistants or support persons must not substitute or have undue influence on the decisions of persons with disabilities, including the expression of their consent. Rationales behind the concept include increased patient autonomy, promotion of coping skills, early help-seeking, avoidance of power struggles, establishment of an asylum function, reduced time spent in inpatient care and prevention of coercive measures. Quantitative data points toward a dramatic reduction of total time spent in inpatient care and of involuntary admissions in patients with previously high inpatient care consumption, whereas qualitative data indicates that the concept increases patient autonomy, responsibility and confidence in daily life. Patient-controlled admission is a promising novel approach to inpatient care in psychiatry. However, available studies are small and quality of evidence is generally low. In this talk an overview of literature review on involuntary admissions and patient autonomy as well as ethical aspects will be given and discussed.

**Disclosure:**

No significant relationships.

